# Effects of Social Connectedness on the Sharing of Employee-Created Content

**DOI:** 10.3389/fpsyg.2022.782304

**Published:** 2022-03-01

**Authors:** Xueting Zhang, Man Chen

**Affiliations:** ^1^Department of Marketing, School of Business, Hunan University, Changsha, China; ^2^Department of Business Administration, School of Economics and Management, China University of Geosciences, Wuhan, China

**Keywords:** employee-created content, content sharing, social connections, social network, promotional content

## Abstract

With the popularity of social network platforms (e.g., Facebook and WeChat), users can easily build social connections with others, create content, and even forward or share content. While previous studies on content sharing shed light on either content creator or receiver, this paper is to investigate whether, when, and how the social connectedness of content creator (i.e., employee) and receiver (i.e., employee’s friend) jointly influence the sharing likelihood of receiver. We conducted a field study on the largest social media platform and two experiments in China. Study 1 found that well-connected receivers prefer to share content from well-connected employee, and poorly connected receivers prefer to share content from poorly connected employee, but if the content contains promotional information, well-connected receivers are less likely to share it from the well-connected employee. Studies 2 and 3 confirmed these findings and verified that self-enhancement motivation acts as a mediator. The findings suggest that firm should choose the “right” employees who will send content to their “right” friends and caution about the crowd-out effect of promotional content. We provide new insights into the joint effects of creator and receiver, the moderating role of promotional content, and the mediating role of self-enhancement, which enriches both viral marketing and social media literature.

## Introduction

With the popularity of social network platforms (e.g., Facebook and WeChat), users can easily build social connections with others, create content, and even forward or share content (Peng et al., 2018). These platforms provide new communication tools for firms. Beyond firms’ official accounts (e.g., brand page at Facebook, Baker et al., 2016; Meire et al., 2019), more and more firms tend to turn their employees into brand champions and encourage them to create content in their personal accounts, which is called *employee-created content* (ECC; Aggarwal et al., 2012). Employee could act as a seed of viral marketing and brand spokesman (Morhart et al., 2009; Zhang et al., 2022). Employee publicly posts content in his or her account, and then, his or her friends receive this content and further share it with their friends and so forth, leading to a widespread of content in the social network. Weber Shandwick company found that 33% of firms encourage their employees to use social media (Shandwick, 2014). Dell, for example, implemented an employee advocacy program that involves 10% of employees on social media in 2015. Finally, more than 10,000 employees use social media to post content and drove 45,000 clicks to its website. However, sometimes ECC is not effective while employee’s friends do not share the content. A total of 54% of brands (e.g., IBM, Adobe, and AT&T) spread content through employees, while only 8% of users share them (Terpening et al., 2016). Therefore, investigating *whether, when, and why* employees’ friends are likely to share content from the employees is critical for the success of viral marketing in social network.

The existing literature has identified several crucial factors that affect receiver’s sharing related to network and content characteristics (see Table 1). Firstly, existing literature studied various types of content, such as positive/negative and informative/persuasive (see Table 1 for details). Among these content, promotional content which contains discount, coupon, or freebies acts as an important catalyst for marketers to spread marketing information (Lee et al., 2018). In practice, many firms offer promotional content to promote consumer engagement and sales (Ryu and Feick, 2007). However, content receivers maybe not be likely to share promotional content with others since such content could hurt the social image and lead to a “crowding-out effect” (Verlegh et al., 2013; Lee et al., 2018). A further investigation on the role of promotional content is needed.

**Table 1:** Selected studies on content sharing.

Study	Role of content creator	Measure of content receiver’s connectedness	Content type	Method	Test mechanism	Main findings
This study	Employee	Degree centrality of employee’s friend	Promotional content	Field data and lab experiments	Yes	Well-connected receivers are more likely to share content from well-connected employees because of self-enhancement. When the content contains promotion, well-connected receivers are less likely to share content from well-connected employees.
Berger and Milkman, 2012	Firm		Positive and negative content	Empirical study and experiments	Yes	Positive content is more viral than negative content, and when the positive or negative content has higher arousal, it will be more viral.
Stephen et al., 2015	Firm		Product-, value-, and brand-related information	Empirical study		Product-related and value-related information has no significant effect on customer’s sharing, while general brand-related information has a positive effect on customer sharing.
Taecharungroj, 2017	Firm		Information sharing, and Action-inducing content	Empirical study		The action-inducing content had the highest average number of retweets and favorites.
Tellis et al., 2019	Firm		Information-focused, Emotion-focused, and Commercial content	Empirical study and experiment		Information-focused content has a significantly negative effect on sharing, except in risky contexts; positive emotion-focused content has a positive impact on sharing; Commercial content hurts sharing.
Hinz et al., 2011	Firm	Participants’ degree centrality		Field experiment, empirical study		Seeding to well-connected and high-betweenness people exhibit a higher response likelihood.
Toubia and Stephen, 2013	User	User’s followers		Field Experiment		Users with a low initial number of followers are more likely to contribute content.
Barasch and Berger, 2014	User	Audience size	Self-presenting content, useful content	Experiments	Yes	Consumers with larger audience sizes prefer to share self-presenting content, whereas consumers with smaller audience sizes prefer to share useful content.

Secondly, most studies on content sharing considered either content creator or receiver. For example, for content receivers, some scholars found that well-connected receivers are more likely to share content (Yoganarasimhan, 2011), others expressed that poorly connected receivers are more likely to share content (Toubia and Stephen, 2013). For content creators, Hinz et al. (2011) found that well-connected people can trigger more followers to share, while Watts and Dodds (2007) expressed that well-connected people are less important as initiators of large cascades of referrals. Furthermore, while some studies have considered the effects of tie strength and mutual connections between content creator and receiver on content sharing (Aral and Walker, 2014; Peng et al., 2018; Nanne et al., 2021), they ignored the social connectedness of both content creator and receiver. Based on communication theory, both information source and receiver determine receiver’s sharing likelihood (Hovland et al., 1953; Berger, 2014). When people receive information from others, they not only care about the type of content but also concern the characteristics of information source, such as network position, social reputation, and identity (Chang and Wu, 2014; Wang et al., 2021).

To resolve the above research gap, we address the following research questions: (1) Whether employee’s friends are likely to share employee-created content in social network? (2) Who (i.e., employee’s friends) is more willing to share content from whom (i.e., employee) and why? (3) Does promotional content enhance or inhibit sharing?

We conducted a field data analysis and two lab experiments. Study 1 analyzed the creation and sharing behaviors of 20,715 individuals. The Cox proportional hazard model revealed that well-connected receivers prefer to share content from the well-connected employee, and poorly connected receivers prefer to share content from the poorly connected employee, but if the content contains promotional information, well-connected receivers are less likely to share it from the well-connected employee. Studies 2 and 3 confirmed these findings and verified that self-enhancement motivation acts as a mediator.

These findings make significant contributions. First, we investigated both the social connectedness of content creator and receiver, while previous research focused on either content creator or receiver. In this paper, we show that the sharing likelihood of receiver depends on both the social connections of content creator and receiver. Second, we find the crowd-out effect of promotional content in social network. Existing studies have analyzed the impact of promotional content on sharing with mixed findings (Taecharungroj, 2017; Lee et al., 2018). We further study the moderating roles of promotional content and show that when well-connected friends read content from a well-connected employee, promotional content will reduce their sharing likelihood. Third, based on the self-enhancement theory, we explained and tested why well-connected receivers are more likely to share content from well-connected employees. Compared with previous studies, such as Hinz et al. (2011) and Peng et al. (2018), they did not test the underlying mechanism.

We also provide detailed, practical suggestions for managers. Firms need to select the “right” employees to send content to the “right” friends (Lanz et al., 2019). Therefore, the appropriate seeding strategy depends not only on the content creator but also on the content receiver. We found that the firm should encourage high(low-)-connected employees to send content to their high(low-)-connected friends. Moreover, if the content contains promotional information, the firm should prevent well-connected employees from sending promotional content to well-connected friends. Otherwise, the sharing could be counterproductive.

## Theoretical Background and Hypotheses Development

### Literature Related to Content Sharing

In recent years, scholars have studied various factors affecting content sharing based on different social media platforms, such as Twitter (Toubia and Stephen, 2013), YouTube (Libai et al., 2013), and Facebook (Lee et al., 2018). Specifically, the first research stream focused on content characteristics (see Table 1). For example, Baker et al. (2016) classified brand content into positive, negative, and neutral, and found that positive content is more viral. Tellis et al. (2019) found that information-focused and commercial content increase sharing, while emotion-focused content inhibits sharing.

Promotional content is the content that contains any forms of monetary and non-monetary promotional information, such as discount, freebie, and coupon (Lee et al., 2018). It is a type of marketing-related information. In practice, many firms often offer various promotional content to attract consumer participation and enhance sales, such as discounts (Wirtz et al., 2013), cash payments (Exley, 2018), and vouchers (Ryu and Feick, 2007), but if the firm cannot accurately target the promotional content to the “right” people, it will increase the marketing cost. Existing studies found that the impacts of promotional content on sharing likelihood could be positive (Wang et al., 2018), negative (Verlegh et al., 2013), or even uncertain (Hong et al., 2017). On the other hand, promotional content can crowd out the people’s image and decrease their sharing intention (Sun et al., 2017). Thus, we will consider whether promotional content is effective.

The second stream centers on social connectedness of users. Well-connected people refer to the people with a high number of connections to others, which is measured by degree centrality (Hinz et al., 2011). The greater the degree centrality, the higher connected the person is in social network. Some scholars indicate that well-connected people (i.e., people with higher degree centrality) are conducive to sharing (Hinz et al., 2011). However, other scholars argue that targeting them is not conducive to sharing (Watts and Dodds, 2007). Besides, most studies considered the social connectedness of either content creator (Barasch and Berger, 2014) or content receiver (Meire et al., 2019). Even though some studies have considered both characteristics of creator and receiver, they addressed network overlap (Peng et al., 2018), tie strength (Tuk et al., 2009), and social distance (Hong et al., 2017) between creators and receivers. In this paper, we consider how social connectedness of both content creators and receivers might influence content sharing.

### Mechanism: Self-Enhancement Motivation

Self-enhancement is the fundamental human motivation for WOM communication (Alexandrov et al., 2013). Sundaram et al. (1998) define self-enhancement motivation as people’s desire to appear smart in front of others, to improve their image. Alexandrov et al. (2013) indicate that self-enhancement motives arise because people want to project a good image to others by sharing information. In general, this motivation implies that people are willing to share information that makes them look good, establishes uniqueness, or helps them gain good social image (Dubois et al., 2016).

Self-enhancement might relate to the number of connections a user has (Toubia and Stephen, 2013; Barasch and Berger, 2014). In practice, the number of connections a person has on social media platforms can provide a signal of the person’s influence or popularity (Chen et al., 2017). Social platforms offer a social stage for people to display themselves, so their sharing on these platforms conventionally should seek to enhance their social status and form good impressions among others. More connections also might arouse such self-enhancement motivations more powerfully, which in turn would affect sharing decisions. For example, people with more social connections might actively share content they receive from certain employees, because such information offers better self-enhancement benefits, as we discuss subsequently.

### Receiver’s Social Connectedness and Content Sharing

In social networks, social connectedness is usually measured by degree centrality or the number of social connections (Goldenberg et al., 2009). Content receiver with a high-degree centrality is well-connected and possess a larger number of connections with others. When receivers have large number of friends, their peer pressure and status considerations might increase (Toubia and Stephen, 2013). Also, based on the self-enhancement theory, well-connected receivers are more concerned about image than poorly connected receivers (Hennig-Thurau et al., 2004). Therefore, when the well-connected people receive content from employees, they will share content more carefully. On the one hand, when well-connected people share content from the employee, they may be seen as “salesman” reducing their social image (Chatterjee, 2015). On the other hand, when receivers have higher number of social connections, the content they shared hardly meets the tastes of all connections. The more connections, the greater the possibility of false matches. Therefore, we suppose that receivers with high-connectedness are less likely to share content from the employee.

*H1:* Well-connected (versus poorly connected) receivers are less likely to share employee-created content.

### Joint Effect of Employee’s and Receiver’s Social Connectedness

Employees are considered to be experts on firm’s product (Eisend, 2004). Besides, the interpersonal nature of employees makes them appear more authentic (Stockman et al., 2017). Based on self-enhancement theory, people can show their social image through content sharing (Hennig-Thurau et al., 2004). In social networks, well-connected people face greater reputational risks, so they are more cautious in sharing to protect their social image (Barasch and Berger, 2014; Chatterjee, 2015). If the well-connected employees are willing to post content, it means that they are willing to stake their reputation, so the reliability of the content appears greater. Besides, content created by a well-connected employee is perceived as more credible (Stockman et al., 2017). When well-connected friends receive content from well-connected employees, sharing can indicate that they have similar tastes, thereby improving receivers’ social image. Therefore, we propose that the well-connected receivers are prone to share content from the well-connected employees.

*H2:*When the content is created by a well-connected employee, well-connected (versus poorly connected) receivers are more likely to share this content (H2a) because of self-enhancement motivation (H2b).

### Moderating Role of Promotional Content

While some scholars found that promotional content can increase customer engagement (Ryu and Feick, 2007; Taecharungroj, 2017), others suggested that promotional content could crowd out people’s self-image (Exley, 2018). Because promotional content (i.e., discounts and freebies) can change the nature of interpersonal communication, once the promotional content is shared; receivers might regard their relationship with content creator as a norm of monetary incentive, not as a friendship link (Wentzel et al., 2014).

Sharing promotional content stimulates receivers’ self-enhancement motivation, due to their suspicion that the creator benefits from sharing that information, which may diminish the credibility of the shared content. Also, promotional content causes receivers to infer less intrinsic motivation and potentially perceive the incentives as the sole driver of recommendation behavior. As we have noted, compared with poorly connected receivers, well-connected receivers tend to be more careful when they worry their sharing might give bad impression to their friends (Tuk et al., 2009; Xiao et al., 2011). Therefore, when well-connected receivers receive promotional content from well-connected employee, it may arouse self-enhancement considerations and lead to more cautious content sharing.

*H3:*Promotional content attenuates the interactive effect of content receiver’s and employee’s social connectedness on sharing likelihood of receiver.

## Overview of Studies

**Figure 1:**
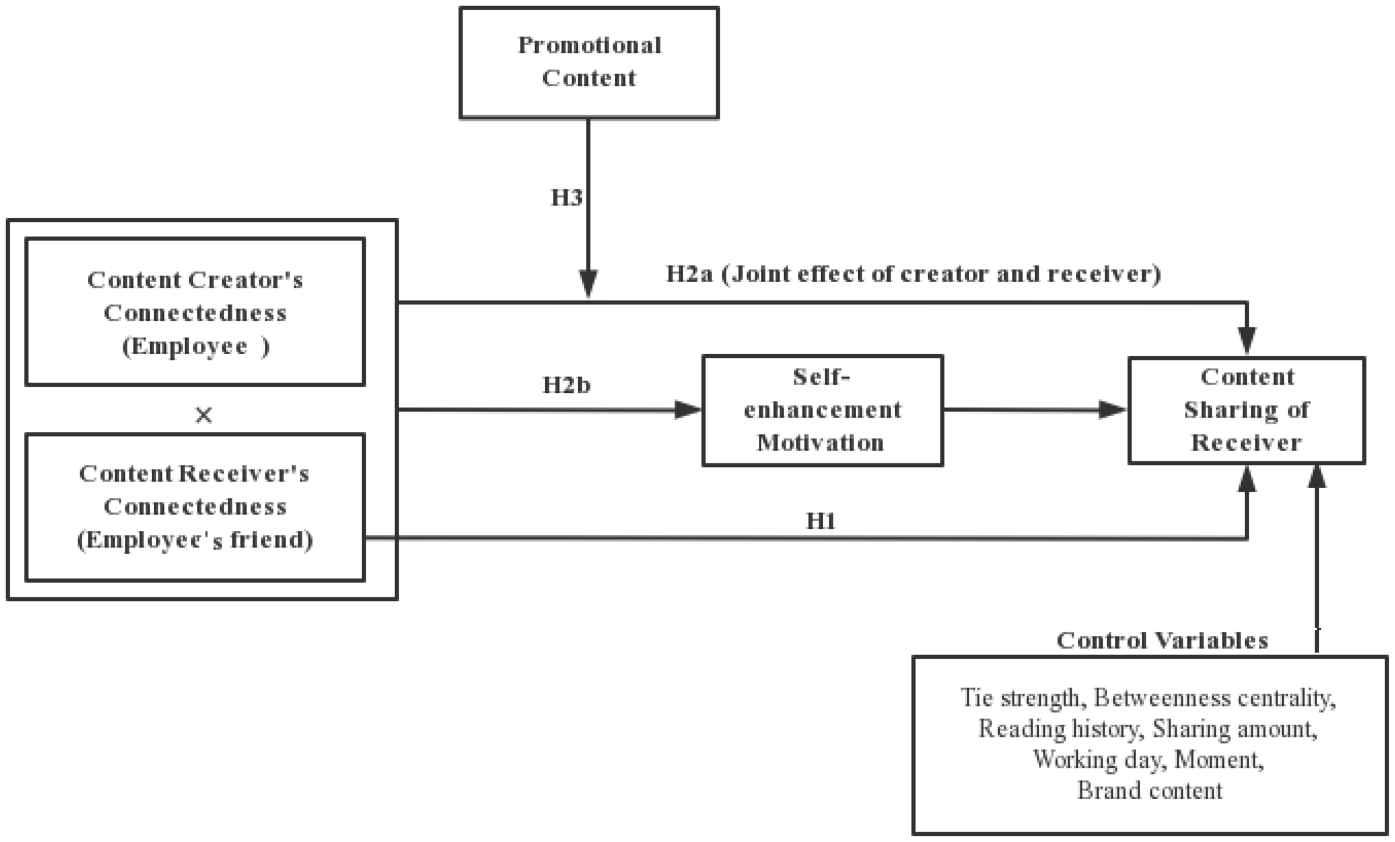
Research framework.

Figure 1 presents our research framework. To test it, we conducted a field data analysis and two lab experiments (Table 2). In Study 1, the field study is to examine the effectiveness of the receiver’s social connectedness on sharing, the interaction between employees’ and receivers’ social connectedness, and the moderating roles of promotional content. Then, with two experimental studies, we replicate the findings of Study 1 in different contexts (restaurant and e-book) and test the underlying mechanisms. Study 2 provides evidence to support H1, H2a, and H2b. Study 3 confirms H2a and H3. In addition, Study 3 also verifies the crowding-out effect of promotional content.

**Table 2:** Overview of studies.

	Study 1	Study 2	Study 3
Hypothesis	H1, H2a and H3	H1, H2a, and H2b	H2a, H3
Method	Field data analysis	Experiment (2 × 2)	Experiment (2 × 2)
Measurement of social connectedness	Degree centrality	Scenario design	Self-report; 1 item
Content type	promotional, non-promotional		promotional, non-promotional
Test Mechanism		Self-enhancement motivation	Crowd-out effect
Product category	Decoration	e-bookstore	Restaurant
Sample	20,715	139	179

## Study 1

### Data and Measurement

The field study was implemented in WeChat, one of the largest social network platforms in China. Users of this platform form undirected friendship network. We cooperated with an anonymous decoration firm, whose main businesses include home decoration design, building materials selection, and selling furniture. With the popularity of social media in China, this decoration firm often uses WeChat to post marketing-related information in its official account and also encourage employees post-content on their personal WeChat accounts. In WeChat, when an employee creates or posts content, her or his friends could receive and read it, and further decide whether to share or forward it. The decoration firm provided us its employees’ content creation and friends’ reading and sharing data from June 2017 to November 2017. During this period, the firm let 4,337 employees post 1,270 marketing-related content on their personal accounts.

The dataset contains information about the actual creation, reading, and sharing behaviors of these employees and their friends. In total, 16,368 employees’ friends formed a total of 327,736 undirected relationships with these employees. Moreover, these contents were forwarded or shared 184,543 times. The dataset also included the title of each content, the nickname of employees and their friends, sharing time, and the number of reads and shares.

Following existing literature, we measure employee’s/receiver’s social connectedness as the degree centrality in social network (Hinz et al., 2011). The degree centrality is defined as the number of actors’ connections (e.g., friends and fans) that are connected to them (Hinz et al., 2011; Ansari et al., 2018). In our study, if there is a direct friendship between individual *i* and *j*, the *a_ij_* = 1. The degree centrality of individual *i* is the total number of friends directly connected to *i*.


$${\mathrm{degree}}_{\left(i\right)}=\underset{i\neq j}{\sum }a_{ij}$$

Next, our dependent variable captures actual content sharing behavior of employees’ friends. For example, on Twitter, the content sharing behavior is referred to as “retweet.” We coded the dependent variable as 1 when an employee’s friend shared the content created from employee, and as 0 when this friend did not share. Then, we coded content that contained promotional information as 1 and 0 otherwise (Lee et al., 2018).

Furthermore, we control the following variables which could influence friend’s sharing likelihood. First, the reading time is coded as 1 if it occurred on a workday and 0 for non-work days (i.e., weekends and legal holidays). Second, the title of content containing brand information is coded as 1 and 0 otherwise. Third, we divided reading moments into two categories, according to whether it was read in the morning. In addition, we also calculated the number of posts that were shared. Following Bond et al. (2012) and Aral and Walker (2014), we controlled the effect of tie strength between content creators (i.e., employee) and receivers (i.e., employee’s friends). Tie strength is measured by the frequency of interaction between them before receivers’ sharing. In addition, we also calculated the betweenness centrality of content receivers. It captures the extent to people who connect two unconnected groups in the social networks (Hinz et al., 2011). The higher the betweenness centrality, the more likely they act as a network intermediary.

### Model Specification

We adopt the hazard modeling method for the survival analysis (Katona et al., 2011; Aral and Walker, 2014). In survival analysis, the occurrence of events is a “failure,” and “survival time” refers to the duration of observation, which is generally recorded from the start time of the event to the last recorded time before the event is invalidated or lost. For this study, “failure” refers to whether the receiver forwards or shares the content during the observation time, and “survival time” refers to the period between the post-time of content and the moment the receiver shares it.

We use a Cox proportional hazards model, noting its advantages. First, the model can process censored data and dynamically identify and measure a variety of factors that influence the user’s forwarding behaviors. Second, the model considers both the event and the time, which a logistic regression cannot do. Third, the model can include multiple factors that influence information sharing. Fourth, the distribution type for survival time is uncertain, but the model does not depend on a particular distribution. Therefore, we can estimate the impact of the employee’s and receiver’s degree centrality on the receiver’s sharing likelihood while controlling for the moderating effect of promotional content:


$$h\left(t,X_{m}\right)=h_{0}\left(t\right)\exp\left(\begin{array}{l} \beta_{1}X_{rdegree}+\beta_{2}X_{edegree}+\beta_{3}X_{promotional}\\ +\beta_{4}X_{rdegree}*X_{edegree}\\ +\beta_{5}X_{rdegree}*X_{edegree}\\ {}*X_{promoitonaal}+\beta_{6}X_{rdegree}\\ {}*X_{promoitional}+\beta_{7}*X_{brand}\\ +\beta_{8}X_{readinghistory}+\beta_{9}X_{moment}\\ +\beta_{10}X_{workday}+\beta_{11}X_{sharingamount}\\ +\beta_{12}X_{betweenness}+\beta_{13}X_{tie\;strength} \end{array}\right)$$

Where $X_{1},X_{2},\ldots X_{m}$ are concomitant variables and the factors that affect forwarding behavior, which include both static and time-dependent factors; $X_{rdegree}$ denotes the receiver’s degree centrality; $X_{edegree}$ represents the employee’s degree centrality; are the partial regression coefficients; and $h_{0}\left(t,\;X_{m}\right)$ is the hazard rate for sharing. If the coefficient of the independent variable is positive, the variable $\;X_{m}\;$ accelerates the occurrence of sharing. If the independent variable’s coefficient is negative, $\;X_{m}\;$ slows down the occurrence of sharing.

### Results and Analysis

Table 3 represents the descriptive statistics. First, it shows that the average number of receivers’ social connections is 37; the average number of creators’ social connections is 146 (i.e., the degree centrality of employees). The maximum value of social connections is 1,866, and the minimum value is 2. Besides, in Table 3, the average variance inflation factor value (VIF) is 1.59, so multicollinearity does not appear to be a concern.

**Table 3:** Descriptive statistics and correlations.

	Mean	SD	VIF	1	2	3	4	5	6	7	8	9	10
1. Receiver’s connectedness	37.08	72.61	3.34	1									
2. Employee’s connectedness	146.27	148.87	1.24	0.04	1								
3. Promotional content	0.32	0.47	1.43	−0.06	−0.33	1							
4. Brand	0.30	0.46	1.06	0.01	−0.18	0.05	1						
5. Reading history	1.44	1.18	1.02	−0.01	−0.05	0.13	0.01	1					
6. Working day	0.72	0.45	1.04	0	−0.04	0.1	−0.1	0.03	1				
7. Moment	0.04	0.19	1.00	0	−0.01	0.02	0.02	0	−0.03	1			
8. Sharing amount	992.45	2884.53	1.30	−0.02	−0.13	0.46	−0.07	0.09	0.16	0.02	1		
9. Betweenness centrality	0.01	0.01	3.33	0.84	0.02	−0.02	0	0	0	0	0	1	
10. Tie strength	2.48	4.62	1.12	0	0.31	−0.18	−0.11	−0.03	−0.05	−0.02	−0.10	0	1

*VIF, variance inflation factor*.

**Figure 2:**
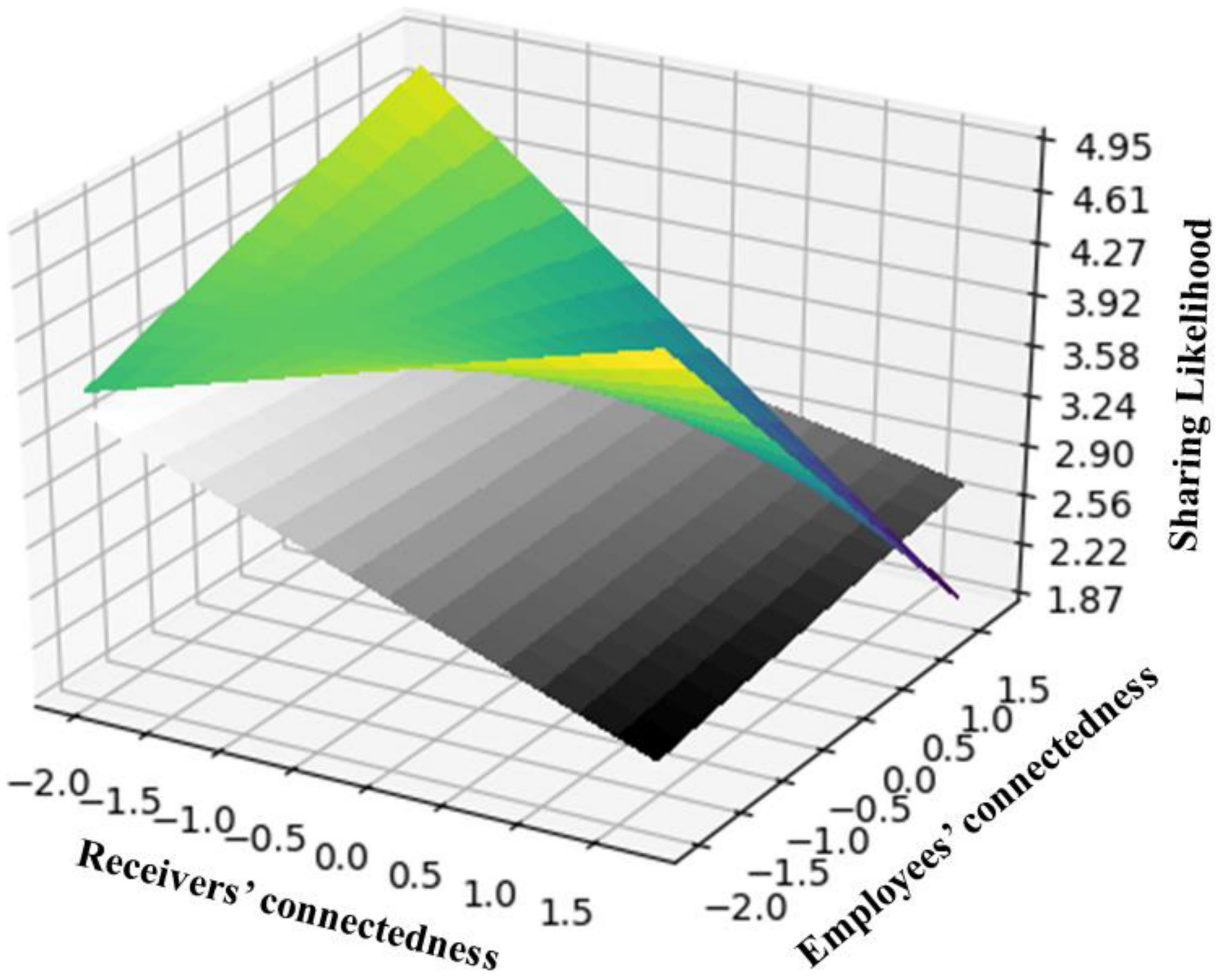
Moderating effect of promotional content. DARK flat indicates the non-promotional condition. Color flat indicates the promotional condition.

Table 4 shows the results of the Cox proportional hazards model. Model 3 adds the moderating effects of employees’ and receivers’ degree centrality, beyond the control variable. The results verified H1, we find a negative effect of receiver’ degree centrality on content sharing ( $\beta_{1}=-.08$ , *p* < 0.01), in support H1. Model 4 adds the promotional content variable and analyzes the moderating effects of promotional content on the relationship between receivers’ and employees’ degree centrality. To support H2a, we find a positive interactive effect of employees’ and receivers’ degree centrality on sharing ( $\beta_{1}=.05$ , *p* < 0.01). The moderating effects of promotional content harm the relationship between receivers’ and employees’ degree centrality ( $\beta_{2}=-.28$ , *p* < 0.01), in support H3. To depict the interactive effect of the employees’ and receivers’ degree centrality for two conditions (i.e., promotional vs. non-promotional), we draw three-dimensional diagrams to show the moderating effect of promotion content in Figure 2. Specifically, the dark flat indicates the non-promotional condition, and the color flat indicates the promotional condition. This three-dimensional diagram indicates that in the condition of non-promotional content, well-connected friends are more likely to share content from well-connected employees. Whereas in the condition of the promotional content, well-connected friends are less likely to share content from the well-connected employee, which again validates H2a and H3.

**Table 4:** Cox proportional hazard regression in Study 1.

Variables		Model 1	Model 2	Model 3	Model 4
		Coef. (S.E.)	Coef. (S.E.)	Coef. (S.E.)	Coef. (S.E.)
Brand		0.26^***^	0.27^***^	0.18^***^	0.15^***^
		(0.02)	(0.02)	(0.02)	(0.02)
Reading history		0.12^***^	0.12^***^	0.11^***^	0.11^***^
		(0.00)	(0.00)	(0.00)	(0.00)
Working day		0.04^*^	0.04^*^	−0.01	−0.08^***^
		(0.02)	(0.02)	(0.02)	(0.02)
Moment		−0.25^***^	−0.25^***^	−0.26^***^	−0.30^***^
		(0.05)	(0.05)	(0.05)	(0.05)
Sharing amount		0.02^**^	0.02^**^	−0.01	−0.11^***^
		(0.01)	(0.01)	(0.01)	(0.01)
Tie strength		−0.62^***^	−0.62^***^	−0.41^***^	−0.32^***^
		(0.03)	(0.03)	(0.03)	(0.03)
Betweenness centrality		−0.05^***^	0.04^*^	0.03	−0.01
		(0.01)	(0.02)	(0.02)	(0.02)
Receiver’s connectedness	**H1**		**−0.10** ^***^	**−0.08** ^***^	**0.01**
			(0.02)	(0.02)	(0.02)
Employee’s connectedness				−0.31^***^	−0.22***
				(0.01)	(0.01)
Receiver’s connectedness × Employee’s connectedness	**H2a**			**0.07** ^***^	**0.05** ^***^
				(0.01)	(0.01)
Promotional content					0.77^***^
					(0.02)
Receiver’s connectedness × Promotional content					−0.29^***^
					(0.04)
Employee’s connectedness × Receiver’s connectedness × Promotional content	**H3**				**−0.28** ^***^
					(0.05)
Number of failures		10,799	10,799	10,799	10,799
Likelihood ratio*χ*^2^		1,939.85	1,963.35	2,558.20	3,806.59
Log likelihood		−105,602.84	−105,591.09	−105,293.66	−104,669.47

^*^p < 0.1, ^**^p < 0.05, and ^***^p < 0.01.

## Study 2

This laboratory experiment has three objectives. First, we repeat the field studies in a more controlled environment to increase validity in the conclusions, and we verify the effect of the receiver’s social connectedness on content sharing of ECC. Second, we verify the interactive effects of the employees’ and receivers’ social connectedness on content sharing. Third, we explore the underlying mechanisms to understand why well-connected receivers are more likely to share content from well-connected employees.

### Procedure

The laboratory experiment was conducted at a university in China. We randomly assigned 139 students (52.52% female, *M*_age_ = 23.52 years) to a 2 × 2 between-subjects designs (receiver’s social connectedness: high vs. low; sender’s social connectedness: high vs. low). To manipulate the receiver’s social connectedness, we showed participants a picture that indicated the number of their friends. The low receiver’s social connectedness condition (21) revealed fewer friends than the high receiver’s social connectedness condition (101). Another picture showed the number of employees’ friends and participants receive the e-book information from the employee. Students are informed that they receive a post related to an e-book from one of their friends who is an employee of an e-book retailer. They are shown a picture and a brief description of this e-book. To check that the manipulation was successful, we asked participants to answer questions about how they perceived the number of friends they had and the employee had (e.g., “What do you think of the number of your connections?” 1 = “not many at all” to 7 = “very many”).

In line with Taylor and Todd (1995), we measured participants’ sharing intention with four questions (“I intend to share the e-book information to my friends more frequently in the future,” “I will try to share the e-book information with my friends,” “I will always make an effort to share the e-book information with my friends,” and “I intend to share the e-book information with my friends”; 1 = “strongly disagree” to 7 = “strongly agree”; Cronbach’s α = 0.95).

Next, we tested the self-enhancement motivation with three items from Dubois et al. (2016): “I shared e-book information so that the message recipient would like me,” “I shared e-book information to create a good impression about myself,” and “I shared e-book information thinking it would have positive consequences on the message recipient’s attitude towards me” (1 = “strongly disagree” to 7 = “strongly agree”; Cronbach’s α = 0.96). We also gathered information about participants’ gender, age, education, income, and sharing frequency in the social network.

### Results and Discussion

A one-way analysis of variance (ANOVA) indicates a significant effect of the receiver’s social connectedness [*F*(1, 137) = 177.01, *p* < 0.01], such that participants in the high-connectedness condition indicated that they possessed more connections (M = 6.45, SD = 0.88) than participants in the low-connectedness (M = 3.73, SD = 1.48). The manipulation of employee’s social connectedness also was successful [*F*(1, 137) = 159.92, *p* < 0.01], in that participants perceived more connections in the condition of high-connectedness of employee (M = 6.33, SD = 1.06) than in the condition of low-connectedness centrality of employee (M = 3.53, SD = 1.53).

**Figure 3:**
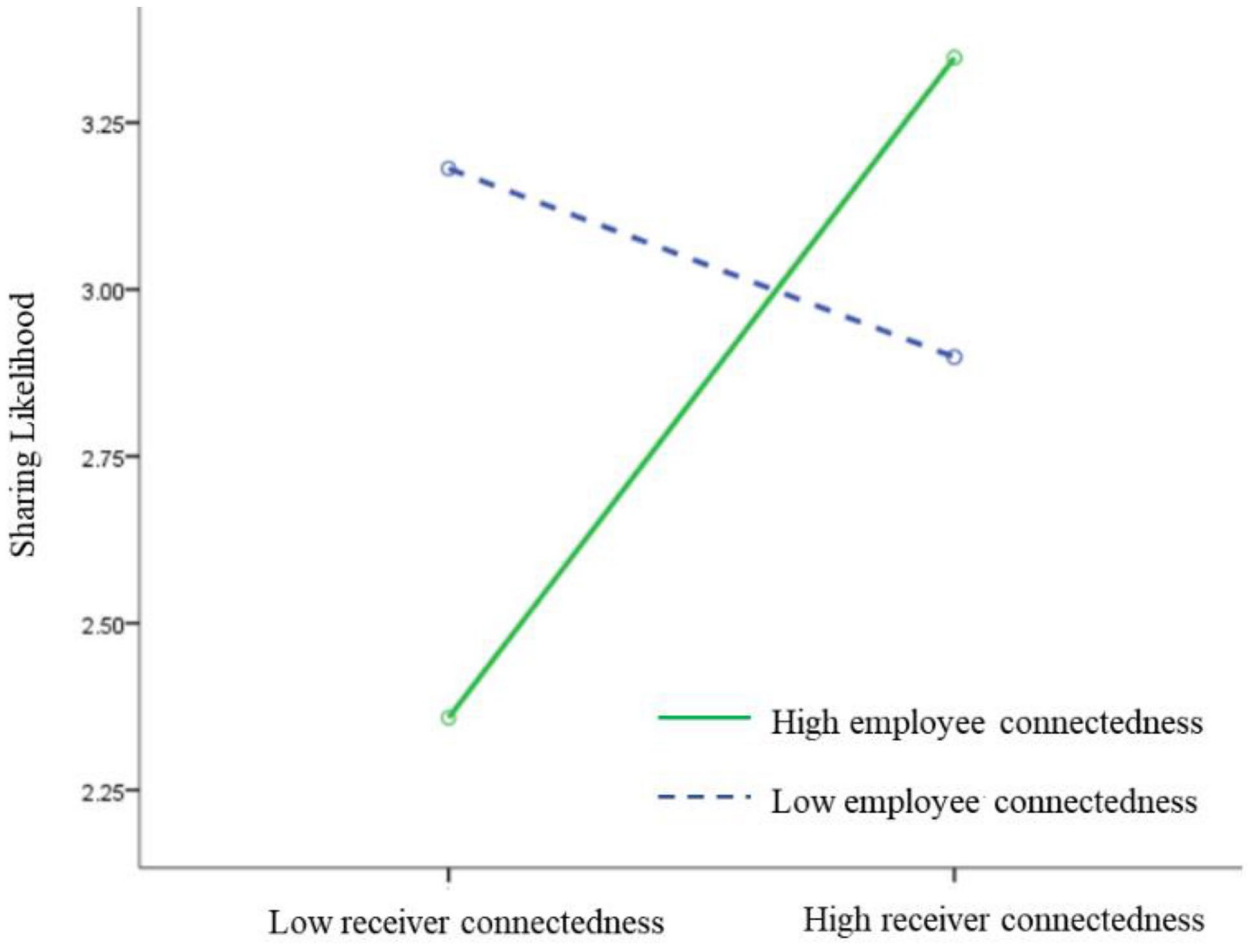
Interaction between employee’s and receiver’s connectedness in Study 2.

The results of the regression analysis in Table 5 indicate a significant negative effect of the receiver’s connectedness on content sharing ( β=−1.64, *p* < 0.05), in support of H1. Besides, we find a significant positive interaction effect of the receiver’s and employee’s connectedness ( β=1.31, *p* < 0.01), in support of H2a. Well-connected receivers are more likely to share content from a well-connected employee. Figure 3 depicts the joint effect of the employee’s and receiver’s connectedness on sharing likelihood. In Figure 3, the solid line represents the condition of the well-connected employee, and the dotted line represents the condition of the poorly connected employee. Figure 3 indicates that under the condition of the well-connected employee, the higher the receiver’s social connections, the greater the likelihood of sharing the content, which verified H2a again.

**Table 5:** Moderating role in Study 2.

Variables	Model 1	Model 2	Model 3
	Coef. (S.E.)	Coef. (S.E.)	Coef. (S.E.)
Number of public accounts	−0.07	−0.07	−0.05
	(0.10)	(0.10)	(0.1)
Viewing frequency	−0.14	−0.15	−0.12
	(0.17)	(0.17)	(0.16)
Sharing frequency	0.33	0.31	0.34
	(0.28)	(0.28)	(0.28)
Gender	0.34	0.35	0.32
	(0.24)	(0.24)	(0.24)
Age	0.24	0.23	0.32
	(0.26)	(0.26)	(0.26)
Education	−0.01	−0.04	−0.09
	(0.25)	(0.25)	(0.24)
Income	−0.08	−0.07	−0.11
	(0.13)	(0.13)	(0.12)
Receiver’s connectedness		0.37	**−1.64** ^**^
		(0.25)	(0.79)
Employee’s connectedness		−0.21	−2.22^***^
		(0.25)	(0.79)
Receiver’s connectedness × Employee’s connectedness			**1.31** ^***^
			(0.49)
F	0.79	0.98	1.65
*R* ^2^	0.20	0.25	0.34

^*^p < 0.1, ^**^p < 0.05, and ^***^p < 0.01.

To examine the mediating role of self-enhancement, we use a bootstrapping method to perform the mediation analysis (Hayes, 2017). The significant index of moderated mediation [*b* = 1.14, SE = 0.40, 95% confidence interval (CI) = (0.37, 1.93)] indicates that self-enhancement mediates the interactive effect of employee’s and receiver’s degree centrality, which supports H2b.

## Study 3

This laboratory experiment has three further objectives. First, we aim to replicate our finding that well-connected receivers are more likely to share content from well-connected employee (H2a) in another industry setting, namely, a restaurant context. Second, we test the moderating role of promotional content (H3). Third, by examining the potential crowding-out effect of promotional content in this study, we explore why well-connected receivers might be unwilling to share promotional content from well-connected employees.

### Procedure

The experiment was conducted at a university in China. We randomly assigned 179 students (44.69% female, *M*_age_ = 21.92 years) to a 2 × 2 between-subjects design (promotional vs. without promotional content; employee’s connectedness: high vs. low), with the receiver’s social connectedness as a measured variable. To start, the students considered a depiction of the number of the employee’s friends. Under the condition of high-degree of employees (151), they have more friends than low-connectedness conditions (21). Next, they were asked to imagine that they received restaurant content from the employee, and we manipulated the promotional content (30% off discount) by providing them with information that either included them or not. To manipulate the senders’ connectedness, the procedure was the same as in Study 2. We also used the same items from Study 2 to measure participants’ sharing willingness (*α* = 0.92) and self-enhancement motives (*α* = 0.91). We collected the same control variables, reflecting participants’ gender, age, education, income, and sharing frequency in the social network.

### Results and Discussion

The manipulation of the employee’s connectedness is successful [*F*(1, 177) = 816.51, *p* < 0.01], such that compared with the low-connectedness of employee condition (M = 1.96, SD = 0.84), participants in a high-connectedness of employee condition perceived that the employee had more connections (M = 5.64, SD = 0.89).

**Figure 4:**
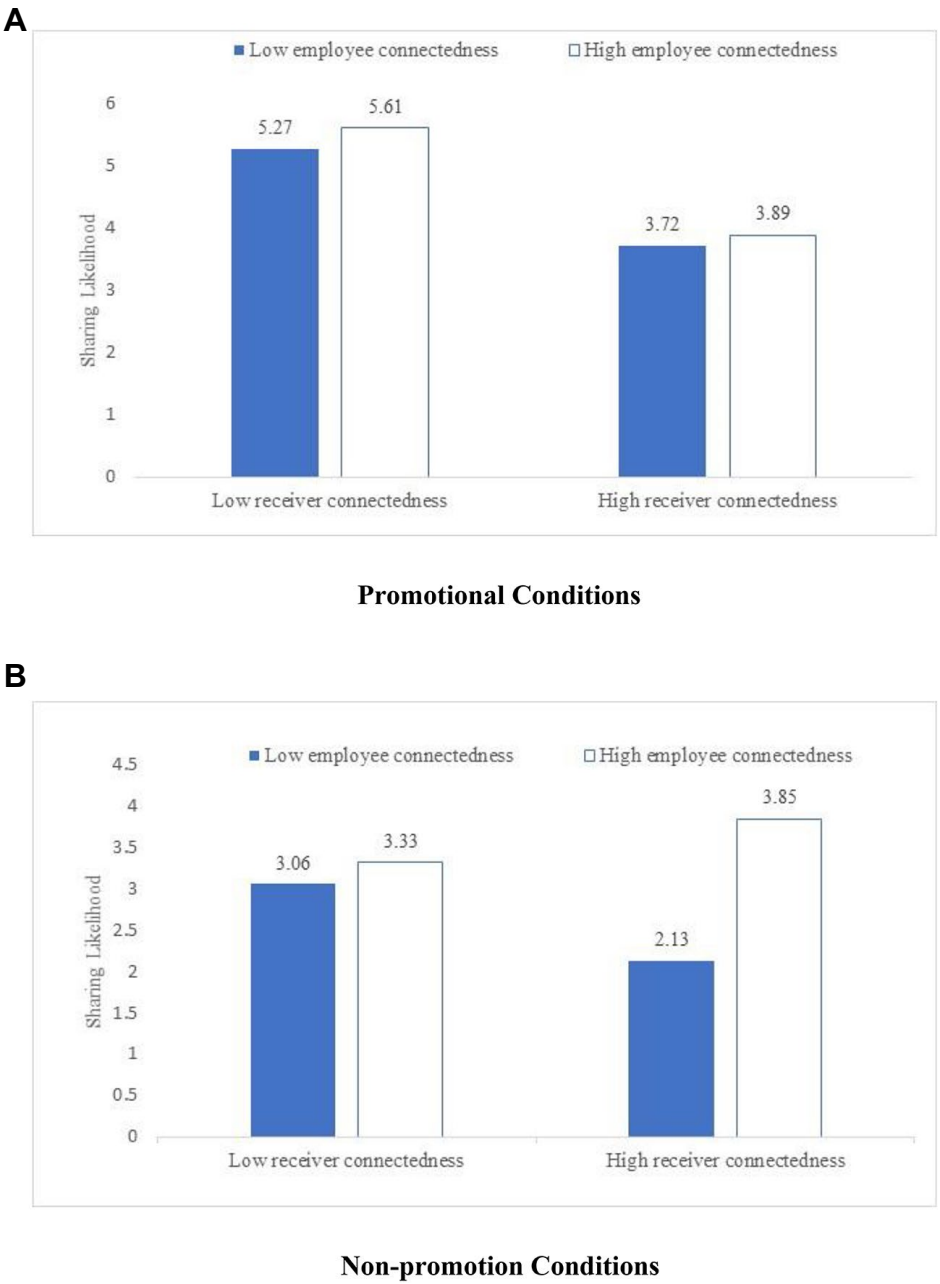
Impact of employee-receiver connectedness on content sharing in Study 3. **(A)** Promotional conditions. **(B)** Non-promotion conditions.

The regression analysis indicates a positive interactive effect of the employee’s and receiver’s connectedness ( β=1.70 , *p < 0*.01). When the information contains promotional, well-connected receivers are less likely to share content from well-connected employees ( β=−.63 , *p* < 0.05). We thus find support for both H2a and H3 (Table 6). In Figure 4, panels a and b illustrate the impact of employees’ and receivers’ connectedness on content sharing in the promotional and without promotional conditions, respectively. Under the condition of non-promotional content, well-connected receivers (M = 3.85) are more likely to share content from the well-connected employees compared to poorly connected receivers (M = 3.33). Under the condition of promotional content, well-connected receivers (M = 3.89) are less likely to share content from the well-connected employees compared to poorly connected receivers (M = 5.61).

**Table 6:** Moderating effect of promotional content in Study 3.

Variables	Model 1	Model 2	Model 3
	Coef. (S.E.)	Coef. (S.E.)	Coef. (S.E.)
Number of public accounts	−0.12	−0.01	−0.03
	(0.12)	(0.1)	(0.10)
Viewing frequency	−0.23	−0.05	−0.12
	(0.19)	(0.17)	(0.16)
Sharing frequency	0.35^**^	0.27^**^	0.29^**^
	(0.16)	(0.13)	(0.13)
Gender	0.19	0.04	0.16
	(0.26)	(0.22)	(0.22)
Age	0.04	−0.20	−0.04
	(0.26)	(0.23)	(0.22)
Education	−0.20	−0.09	−0.04
	(0.17)	(0.15)	(0.14)
Income	−0.05	0.03	−0.13
	(0.26)	(0.23)	(0.22)
Receiver’s connectedness		**−0.91** ^***^	**−1.30**
		(0.22)	(1.14)
Employee’s connectedness		0.61^***^	−0.55
		(0.23)	(0.67)
Promotional content		1.52^***^	3.62^***^
		(0.23)	(0.66)
Receiver’s connectedness × Promotional content			−0.50
			(0.61)
Receiver’s connectedness × Employee’s connectedness			**1.70** ^***^
			(0.61)
Receiver’s connectedness × Employee’s connectedness × Promotional content			**−0.63** ^**^
			(0.28)
*R* ^2^	0.22	0.57	0.63
*F*	1.22	7.86	8.23

^*^p < 0.1, ^**^p < 0.05, and ^***^p < 0.01.

**Figure 5:**
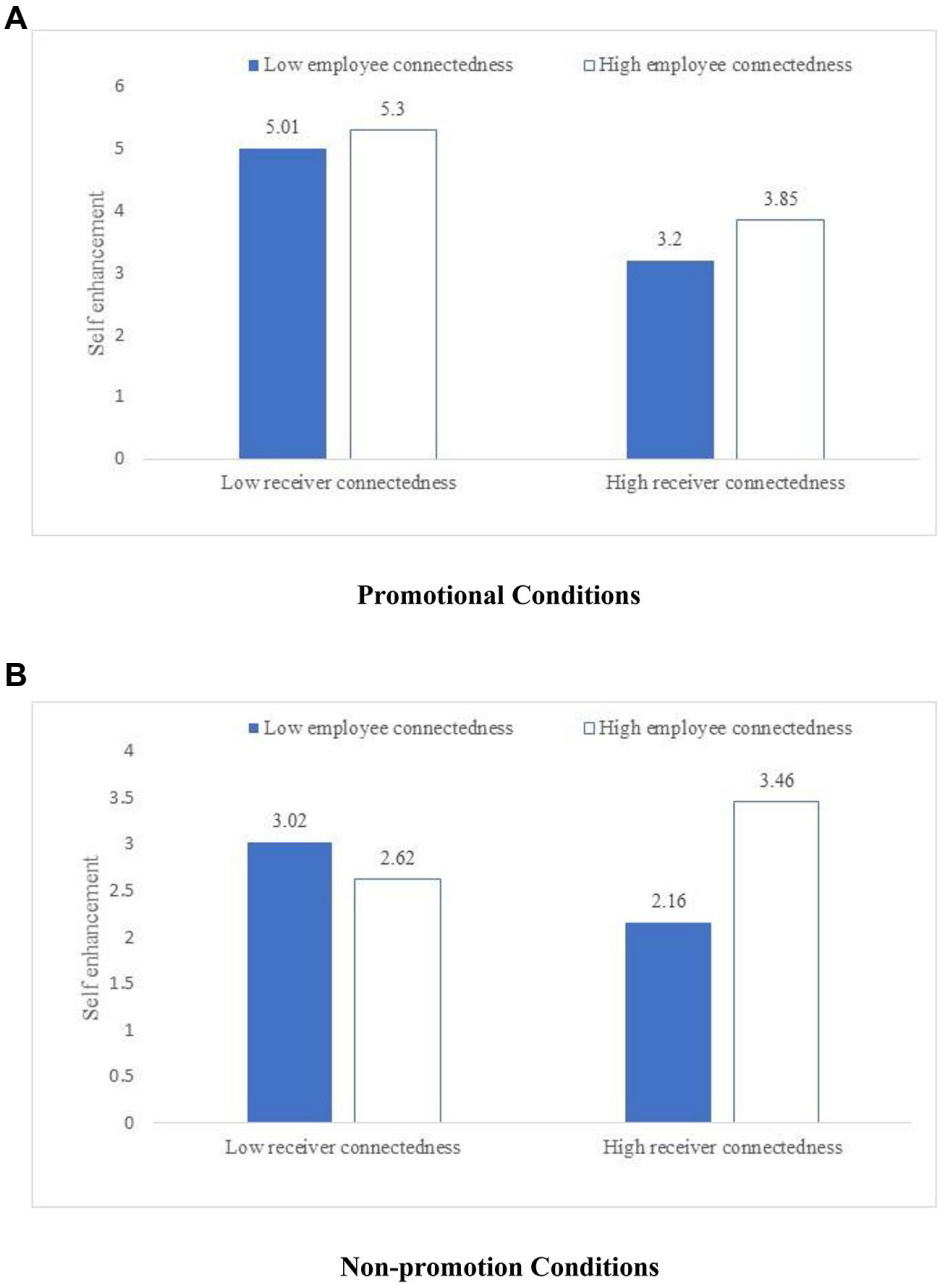
Crowd-out effect of promotional content in Study 3. **(A)** Promotional conditions. **(B)** Non-promotion conditions.

We also test for a crowding-out effect of promotional content. First, we divide the sample into two groups: promotional or without promotional content. Second, we include self-enhancement as a dependent variable and the employee’s and receiver’s connectedness as independent variables in an ANOVA. Figure 5 shows the crowd-out effect of promotional content on self-enhancement motivation. Specifically, under the condition of non-promotional content, the self-enhancement motivation of the well-connected receiver (M = 3.46) is higher than that of the poorly connected receiver (M = 2.62) when the content is from the well-connected employee. But under the condition of promotional content, the self-enhancement motivation of the well-connected receiver (M = 3.85) is lower than that of the poorly connected receiver (M = 5.3) when the content is from the well-connected employee. That is to say that promotional information crowds-out people’s self-enhancement. In sum, Study 3 verifies H2a and H3 and provides additional support for the crowd-out effect of promotional content.

## General Discussion and Implications

Although many firms attempt to seed viral campaigns by encouraging well-connected people to disseminate their marketing information, a high failure rate plagues managers and causes controversy in academia (Watts and Peretti, 2007). We explore a new seeding strategy from the perspective of employees. With one field data and two lab experiments, we identify managerially relevant boundary conditions related to social connectedness and promotional content. The studies indicated that well-connected receivers (versus poorly connected) are less likely to share employee-created content, while they prefer to share content from well-connected employees and poorly connected receivers prefer to share content from the poorly connected employee, but if the content contains promotional information (e.g., discount and coupon), well-connected receivers are less likely to share it from the well-connected employee.

### Theoretical Implications

Our research contributes to marketing literature in several ways. First, according to communication theory (Hovland et al., 1953), in addition to personal characteristics, information sources are important determinants of information diffusion. However, extant content marketing literature mostly focuses on personal network characteristics, without considering the role of the information source (Libai et al., 2013). In this paper, we note the social connectedness of information sources as a driver of content sharing, not just the information receiver’s social connectedness. Besides, existing findings on the impact of social connectedness on sharing are controversial (Watts and Dodds, 2007; Hinz et al., 2011). Using a field study and lab experiments, we verify that the information source constitutes an important boundary condition. Specifically, well-connected receiver is likely to share information from the well-connected employee, and poorly connected receiver is likely to share information from poorly connected employee.

Second, we explore the underlying mechanism that explains why mismatches in the number of connections between content creators and receivers lead to different effects (Hinz and Spann, 2008). Following self-enhancement theory, we find that well-connected people are more likely to share content from well-connected employees because it stimulates their self-enhancement motivation. As a result, our conclusions enrich both content marketing and social network literature.

Third, this paper provides some new insights into the moderating effects of promotional content. Extant studies of information sharing often consider social network characteristics, and they also report mixed findings of the effectiveness of promotional content for encouraging content sharing (Ryu and Feick, 2007; Hong et al., 2017). Our finding of the interaction between promotional content and social connectedness suggests a new perspective on previous findings. Furthermore, we verify the crowding-out effect of promotional content in social network. Well-connected people who receive promotional content from well-connected employees experience weakened self-enhancement motivations, which decrease their sharing likelihood.

### Managerial Implications

This paper provides several implications for managers who want to design effective seeding campaigns and content marketing. First, firms can use sociometric data to improve their effectiveness of marketing campaigns and select the optimal seeding. Particularly, the firm could encourage its employees to post-content on social media, which will facilitate the spread of marketing-related information. However, firms need select the “right” employees who will post-content to the “right” friends (Dost et al., 2018). That is, firms need match the degree centrality of employees and employees’ friends to achieve the wide diffusion of content. In particular, well-connected employees should spread content to their well-connected friends.

Second, our research provides firms with more precise promotional strategies in the social networking environment. Especially, we found that the promotional content can be effective and backfire. On the one hand, the firm can leverage the promotional content to stimulate content sharing, especially if well-connected employees spread promotional content to their less-connected friends. Although past research has established that promotional content can increase receivers’ sharing intention, it has not taken the impact of social network characteristics into account (Kornish and Li, 2010; Hong et al., 2017). Our research provides suggestions for developing more precise promotional strategies in social networks that can reduce firms’ advertising costs.

Third, our findings also caution about the potential backfiring effects of promotional content. When firms encourage employees to spread promotional content, they must balance those employees’ and their followers’ social connections. Specifically, promotional content can crowd out people’s image effects. Therefore, if firms decide to disseminate promotional content, they should avoid allowing well-connected employees to disseminate promotional content to well-connected friends, as promotional content can crowd out friends’ image.

Fourth, our research has identified an important psychological mechanism of self-enhancement motivation. Therefore, when firms disseminate their marketing messages, they should pay attention to the motivation of information receivers, which will help the success of marketing campaigns. Specifically, when well-connected friends receive information from well-connected employee, their self-enhancement motivation will increase, thus improving sharing intention. However, promotional content can undermine friends’ self-enhancement motivation. Overall, our findings provide important managerial implications for firms.

### Limitations and Further Research

This research offers one of the first empirical demonstrations of a link between promotional content and social network characteristics. However, our data are limited to Chinese social network platform. Further research could verify the generalizability of the conclusions in other cultural and national settings (e.g., Facebook in the United States). Furthermore, we assume the social network is static. Further research could capture the features of network dynamics and also investigate potentially influential factors, such as tie strength and product type (Park et al., 2018).

## Data Availability Statement

The raw data supporting the conclusions of this article will be made available by the authors, without undue reservation.

## Author Contributions

XZ assisted in research, collected and analysed data, and wrote earlier version of the manuscript. MC designed the studies and revised the manuscript. All authors contributed to the article and approved the submitted version.

## Conflict of Interest

The authors declare that the research was conducted in the absence of any commercial or financial relationships that could be construed as a potential conflict of interest.

## Publisher’s Note

All claims expressed in this article are solely those of the authors and do not necessarily represent those of their affiliated organizations, or those of the publisher, the editors and the reviewers. Any product that may be evaluated in this article, or claim that may be made by its manufacturer, is not guaranteed or endorsed by the publisher.
